# Relationship between insulin-like growth factor axis gene polymorphisms and clinical outcome in advanced gastric cancer patients treated with FOLFOX

**DOI:** 10.18632/oncotarget.9100

**Published:** 2016-04-29

**Authors:** Sung Yong Oh, Aesun Shin, Seong-Geun Kim, Jung-Ah Hwang, Seung Hyun Hong, Yeon-Su Lee, Hyuk-Chan Kwon

**Affiliations:** ^1^ Department of Internal Medicine, Dong-A University College of Medicine, Busan, Korea; ^2^ Department of Preventive Medicine, Seoul National University, Korea; ^3^ Department of Internal Medicine, Pusan National University Yangsan Hospital, Yangsan, Korea; ^4^ Cancer Genomics Branch, Research Institute, National Cancer Center, Goyang, Gyeonggi-do, Korea; ^5^ Sillajen Inc., Busan, Korea

**Keywords:** insulin-like growth factor, polymorphism, gastric cancer

## Abstract

The insulin-like growth factor (IGF) axis plays a crucial role in proliferation, differentiation, migration, angiogenesis, and apoptosis. The present study evaluated the associations between IGF axis single-nucleotide polymorphisms (SNPs) and clinical outcomes in advanced gastric cancer (AGC) patients treated with oxaliplatin, 5-fluorouracil, and leucovorin (FOLFOX). A total of 190 patients undergoing FOLFOX chemotherapy for AGC were considered eligible for this study. Forty-four SNPs of 10 IGF axis genes were genotyped. Levels of serum IGF1 were measured using enzyme-linked immunoassays. SNPs of the IGF1R (rs12423791), and IGF1 (rs2162679, rs5742612, rs35767) genes were significantly associated with tumor response to FOLFOX. SNPs of rs4619 and rs17847203 were significantly associated with PFS (hazard ratio [HR] 0.575, 95% CI 0.385–0.858, *P* = 0.007; and HR 2.530, 95% CI 1.289–4.966, *P* = 0.007; respectively). SNPs of rs2872060 were significantly associated with OS—OS was shorter in patients carrying the TT variant than in those with the GG/GT genotypes (HR, 1.708, 95% CI 1.024–2.850, *P* = 0.040). The GT genotype of rs12847203 was also identified as an independent prognostic factor (HR 2.087, 95% CI 1.070–4.069, *P* = 0.031). These results suggest that IGF axis-pathway SNPs could be used as prognostic biomarkers of the outcome of FOLFOX chemotherapy in AGC patients. This information may facilitate identification of population subgroups that could benefit from IGF1R-targeted agents.

## INTRODUCTION

Gastric cancer remains an important health problem despite its declining incidence in the west. An estimated 951,600 new gastric cancer cases and 723,100 deaths occurred in 2012 [1]. Although the incidence of gastric cancer among Koreans has decreased over the past two decades, it is the most common carcinoma in males, and the third most common in females, and is the third-leading cause of cancer-related death in Korea [2]. The prognosis of patients with advanced gastric cancer (AGC) remains poor; chemotherapy confers only a minimal survival advantage (median survival, < 12 months) [3]. Development of more-effective chemotherapeutic drugs and regimens is needed.

The folinic acid/5-fluorouracil/oxaliplatin combination (FOLFOX) has proven to be an effective first- or second-line treatment regimen for AGC [4, 5]. FOFOX regimen showed similar clinical effects, and relatively little toxicities compared to other regimens in AGC [3].

The FOFOX regimen shows similar clinical efficacy, and less toxicity, compared to other regimens for AGC [3]. There is increasing demand for improved techniques for the prediction of treatment response and survival, which may facilitate customized chemotherapy and result in significantly enhanced survival rates.

The insulin-like growth factor (IGF) axis is composed of two peptide ligands (IGF1 and IGF2), two cell-surface receptors (IGF1R and IGF2R), six specific IGF-binding proteins (IGFBP1 to IGFBP6), and proteins involved in intracellular signaling, such as the insulin-receptor substrate (IRS) family (IRS1- 4) [6]. The IGF axis-signaling pathway affects tumor biology via both metabolic and mitogenic pathways. The IGF1 gene encodes a protein similar in function and structure to insulin. Additionally, IGF1 affects tumor cell proliferation via the RAS-RAF- MAP kinase signaling pathway and also has antiapoptotic effects mediated by the phosphatidylinositol-3 kinase/AKT pathway, which ultimately activates downstream transcription factors that regulate the expression of proliferative, differentiation, and antiapoptotic factors [7].

Consistent data indicate the role of IGFs in the development [8], the progression [9] and sensitivity to chemotherapy [10] of gastric cancer. Previously, we have attempted to assess the association between serum levels of IGFs and clinical outcomes of AGC, but found no significant correlations [11]. To date, limited published data on the associations of IGF polymorphisms with gastric cancer prognosis are available, and those extant are discrepant [9, 10].

Recent studies provide evidence of associations between poor clinical outcomes with genotypes of gastric cancer.

Here, we investigated the relationship of single nucleotide polymorphisms (SNPs) of IGF-axis genes with the clinical outcomes of AGC patients treated with FOLFOX. The results reveal the associations of IGF-axis gene SNPs with clinical outcomes of AGC patients treated with first-line FOLFOX palliative chemotherapy.

## RESULTS

### Patient characteristics

A total of 190 patients were enrolled. Demographic details of the patients are shown in Table 1. The patients consisted of 125 males and 65 females, and their median age was 55 years (range 24–79 years). Ninety-seven patients underwent curative operation before the onset of metastasis (stage I, *n* = 8; stage II, *n* = 28; stage III, *n* = 41), and palliative resection was performed in 30 stage IV patients. Seventy-nine patients (41.6%) received 5-FU-based adjuvant chemotherapy. Almost all patients had a good performance status. No significant association between the SNP genotypes and patient characteristics was detected (data not shown).

**Table 1:** Patients' characteristics

		number	%
Sex	Male	125	65.8
	Female	65	34.2
Age	Median	55 years	
	Range	(24–79 years)	
ECOG performance status	0,1	186	97.9
	2	4	2.1
Lauren classification	Intestinal	26	13.7
	Diffuse	41	21.6
	Mixed	18	9.5
	Unknown	105	55.3
Initial stage	1	8	4.2
	2	28	14.7
	3	41	21.6
	4	113	59.5
Operation	+	127	66.8
	–	63	33.2
Adjuvant therapy	+	79	41.6
	–	111	58.4
		
No. of metastasis	1	106	55.8
	2	54	28.4
	> 3	30	15.8
CEA	< 5 ng/ml	119	62.6
	≥ 5 ng/ml	54	28.4
	Unchecked	17	8.9

CEA, carcinoembryonic antigen; ECOG, eastern cooperative oncology group.

Genotyping for the 44 IGF axis gene polymorphisms was determined for all 190 patients by a researcher blinded to the clinical status of the patients. Among the 53 SNPs selected (IGF1, *n* = 17; IGF1R, *n* = 11; IGF2, *n* = 2; IGF2R, *n* = 4; IGFBP1, *n* = 1; IGFBP3, *n* = 4; IGFBP5, *n* = 2; IRS1, *n* = 5; IRS2, *n* = 6; and IRS4, *n* = 1), data for nine (rs10735380, rs1063599 in IGF1, rs11042751 in IGF2, rs2854746, rs2854744 in IGFBP3, rs1801278 in IRS1, and rs1974134, rs12853546, rs1805097 in IRS2) could not be generated. All genotype frequencies did not deviate from the Hardy-Weinberg equilibrium, as the cut-off value was a *P*-value < 0.05 by chi-squared test.

### IGF-axis genotype and chemotherapy response

The overall chemotherapy response rate was 34.2% (95% CI: 20.0–40.5%). Six patients achieved complete responses (3.1%), 59 patients achieved partial responses (31.1%), 76 patients showed a stable disease (40.0%) and 49 showed a progressive status (25.8%). Lauren's classification (*P* = 0.029) and number of metastases were related to the response to chemotherapy (*P* = 0.034). Other parameters–including gender, age, previous operation, initial stage, adjuvant chemotherapy, and carcinoembryonic antigen (CEA) level–were not significantly correlated with the clinical response to FOLFOX chemotherapy.

IGF-axis SNPs and their associations with chemotherapy responses are shown in Table 2. Several SNPs of the IGF1R (rs12423791), and IGF1 (rs2162679, rs5742612, rs35767) genes were significantly associated with tumor response. None of the other analyzed SNPs were predictive of the response to FOFLOX treatment. Correlations of IGF-axis genotypes that were related to chemotherapy response with serum levels of IGF1 are shown in Table 2. None of the tested SNPs was associated with serum IGF1 level (data not shown).

**Table 2:** Treatment response and serum IGF1 level according to genotype of insulin-like growth factor axis genotype

Locus	Genotype	ORR*	%	*P*	IGF1 (ng/ml)^#^	*P*
IGF1 rs4764887	AA	5/8	62.5	0.051	34.9 ± 29.5	0.790
	AG	19/75	25.3		40.5 ± 30.4	
	GG	40/107	37.4		36.2 ± 32.0	
IGF1R rs12423791	CC	5/6	83.3	**0.010**	32.1 ± 33.3	0.820
	CG	21/81	25.9		39.9 ± 29.3	
	GG	38/103	36.9		36.6 ± 32.7	
IGF1 rs2162679	AA	33/81	40.7	**0.019**	35.6 ± 30.7	0.841
	AG	22/92	23.7		39.4 ± 33.2	
	GG	9/17	52.9		39.0 ± 37.8	
IGF1 rs5742612	CC	7/9	77.8	**0.016**	23.9 ± 13.5	0.108
	CT	24/86	27.9		45.2 ± 33.5	
	TT	33/62	53.2		37.8 ± 31.1	
IGF1 rs35767	CC	33/82	40.2	**0.005**	35.1 ± 30.5	0.597
	CT	22/93	23.7		41.0 ± 33.3	
	TT	9/15	60.0		32.5 ± 18.6	

* by Fisher's exact and chi-square test;

# mean ± standard deviation; ORR, overall response rate.

### Association of IGF-axis genotype with survival

The median duration of follow-up was 14.6 months (range, 1.0–48.3 months). The PFS was 4.5 months (95% CI 3.8–5.1 months), and the median OS was 12.9 months (95% CI 10.6–15.2 months). Among the clinical parameters evaluated, gender, previous operation, Lauren's classification, adjuvant chemotherapy and CEA were not correlated with either PFS or OS. However, patient age was related to both PFS (*P* = 0.035) and OS (*P* = 0.011), such that younger patients (< 60 years of age) had better clinical outcomes.

**Figure 1:**
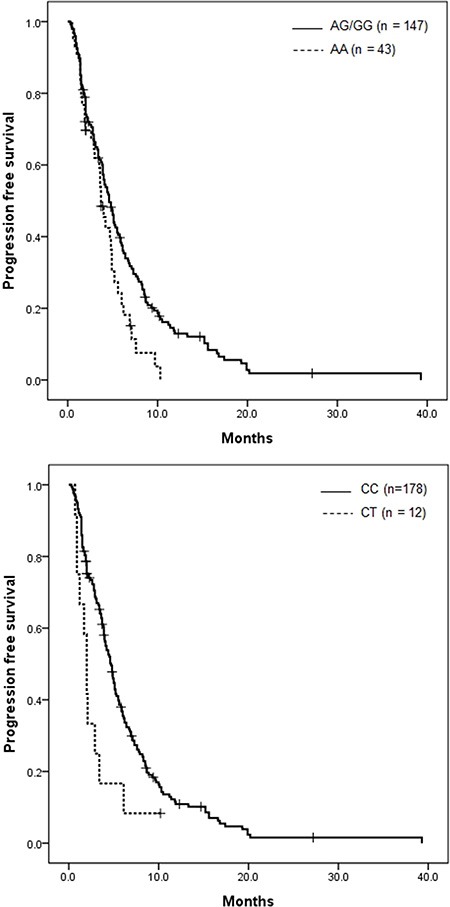
Kaplan-Meier progression-free survival curve according to IGF-axis gene polymorphism (**A**) IGFBP1 rs4619 (*P* = 0.021) and (**B**) IGF1R rs17847203 (*P* = 0.010).

**Figure 2:**
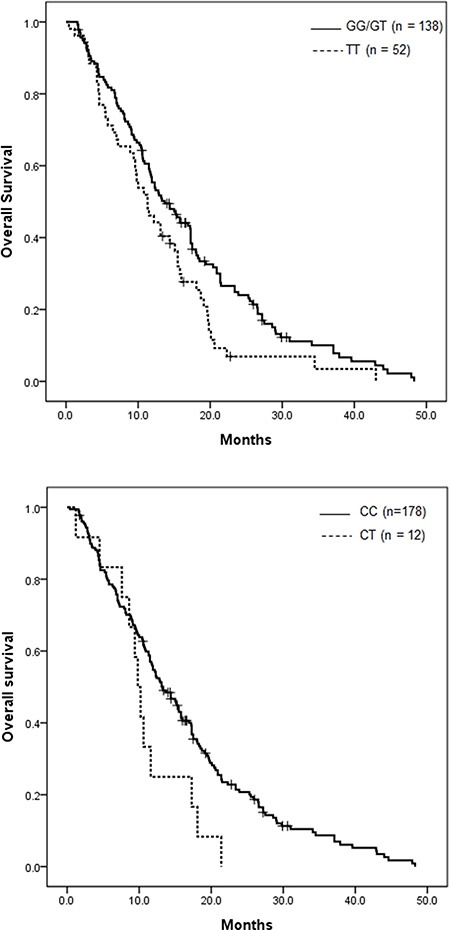
Kaplan-Meier overall survival curve according to IGF-axis gene polymorphism (**A**) IGF1R rs2872060 (*P* = 0.022) and (**B**) IGF1R rs17847203 (*P* = 0.046).

Table 3 shows the association of IGF-axis SNPs with PFS and OS in the 190 patients analyzed. In univariate analysis, patients with the IGFP1 rs4619 polymorphism AG/GG genotype had a longer PFS of 4.6 months, compared with 3.7 months for those with the AA genotype (*P* = 0.021, Figure 1A). Also, patients with the IGF1R rs17847203 CC genotype showed better PFS than those with the CT genotype (4.7 vs. 2.0 months, *P* = 0.010, Figure 1B). Among the investigated IGF-axis SNPs, five IGF1R polymorphisms—rs7166558, rs2229765, rs12437963, rs2872060, rs17847203—were significantly related to OS (*P* = 0.023, 0.011, 0.021, 0.022 and 0.046, respectively) by the dominant model. The OS curves of IGF1R rs2872060 and IGF1R rs17847203 are shown in Figure 2A and 2B, respectively.

**Table 3:** Association of insulin-like growth factor axis genotype with progression free survival and overall survival

Locus	Genotype	No. of patients	PFS (mo)	*P*	OS (mo)	*P*
IGFBP1 rs4619	AA	44	3.7	**0.039**	10.3	0.406
	AG	95	4.9		13.7	
	GG	51	4.6		15.5	
	AA/AG vs GG	138/52	4.5/4.4	0.965	12.4/15.5	0.815
	AA vs AG/GG	43/147	3.7/4.6	**0.021**	10.7/14.4	0.209
IGF1R rs7166558	AA	81	4.2	0.716	11.7	**0.038**
	AG	84	4.5		12.8	
	GG	25	5.6		17.3	
	AA/AG vs GG	165/25	4.4/5.6	0.414	12.2/17.3	**0.023**
	AA vs AG/GG	81/109	4.2/4.6	0.776	11.7/13.7	**0.046**
IGF1R rs2229765	AA	27	5.6	0.687	17.3	**0.037**
	AG	86	4.5		12.3	
	GG	77	4.2		11.9	
	AA vs AG/GG	27/163	5.6/4.4	0.410	18.1/11.9	**0.011**
	AA/AG vs GG	113/77	4.6/4.2	0.588	13.2/11.9	0.115
IGF1R rs12437963	AA	72	4.2	0.676	11.5	**0.044**
	AG	87	4.8		12.9	
	GG	31	4.0		17.2	
	AA/AG vs GG	159/31	4.5/4.0	0.915	12.3/17.2	0.104
	AA vs AG/GG	72/118	4.2/4.7	0.385	11.5/14.1	**0.021**
IGF1R rs2872060	GG	54	3.5	0.220	12.9	0.072
	GT	84	5.1		14.4	
	TT	52	4.5		11.3	
	GG/GT vs TT	138/52	4.4/4/5	0.240	13.7/11.3	**0.022**
	GG vs GT/TT	54/136	3.5/4.9	0.402	12.9/12.8	0.478
IGF1R rs17847203	CC	178	4.7	**0.010**	13.2	**0.046**
	CT	12	2.0		9.8	

PFS, progression free survival; OS, overall survival; mo, months.

Cox proportional hazards regression models of the six SNPs and clinicopathologic features are shown in Table 4. In the multivariate analysis, number of metastases (HR 1.274, 95% CI 1.034–1.568, *P* = 0.023) remained independent prognostic factors for PFS. Patient age (HR 1.501, 95% CI 1.082–2.083, *P* = 0.015), and previous operation (HR 1.756, 95% CI 1.202–2.564, *P* = 0.004) were significantly related to OS. Two IGF-axis gene polymorphisms, IGF1 rs4619 and IGF1R rs17847203, were significantly associated with PFS (HR 0.575, 95% CI 0.385–0.858, *P* = 0.007; HR 2.530, 95% CI 1.289– 4.966, *P* = 0.007; respectively). IGF1R rs2872060 was associated with OS after adjustment for demographic and clinicopathologic factors, showing shorter OS in patients carrying the TT variant compared to GG/GT (HR, 1.708, 95% CI 1.024–2.850, *P* = 0.040). Interestingly, the GT genotype of IGF1R rs17847203 was also identified as an independent prognostic factor (HR 2.087, 95% CI 1.070– 4.069, *P* = 0.031) for OS in addition to for PFS. Other investigated polymorphisms of the IGF-axis gene were not associated with either PFS or OS.

**Table 4:** Multivariate analysis

	Progression Free Survival			Overall Survival		
	HR	95% CI	*P* value	HR	95% CI	*P* value
Age (< 60 or ≥ 60)	1.361	0.973–1.904	0.072	1.501	1.082–2.083	**0.015**
Operation (yes or no)	1.121	0.784–1.603	0.531	1.756	1.202–2.564	**0.004**
Type (diffuse or intestinal)	1.095	0.901–1.331	0.363	0.941	0.769–1.151	0.552
Number of metastasis (1 or > 1)	1.274	1.034–1.568	**0.023**	1.042	0.843–1.289	0.703
IGFBP1 rs4619 (AA or AG/GG)	0.575	0.385–0.858	**0.007**	0.707	0.484–1.031	0.072
IGF1R rs7166558 (AA/AG or GG)	0.809	0.181–3.617	0.782	1.874	0.245–14.31	0.545
IGF1R rs2229765 (AA or AG/GG)	1.004	0.241–4.183	0.996	2.563	0.347–18.93	0.356
IGF1R rs12437963 (AA or AG/GG)	1.114	0.694–1.789	0.655	1.139	0.705–1.843	0.595
IGF1R rs2872060 (GG/GT or TT)	1.379	0.849–2.240	0.195	1.708	1.024–2.850	**0.040**
IGF1R rs17847203 (CC or CT)	2.530	1.289–4.966	**0.007**	2.087	1.070–4.069	**0.031**

The combined effects of polymorphisms in five SNPs of IGF1R were analyzed (Supplementary Table 3). None of the combination of risk alleles showed a statistically significant association with PFS.

## DISCUSSION

IGF1 and its binding proteins play key roles in the genesis of many types of cancer [12]. Individual genetic variations in the IGF1 signaling pathway have been associated with the prognosis of several common cancers. A 3′UTR polymorphism in IGF1 predicts survival of Chinese non-small cell lung cancer patients [13]. SNPs in the IGF axis may be related to inter-individual variation in the risk and progression of pancreatic cancer, and its resistance to treatment [14].

Since few preliminary data are available, the significance of genetic factors of the IGF axis in AGC remains undefined. IGF1R expression was a significant predictor of poor survival in patients with AGC [15]. It was also related to poor survival in AGC after curative resection and adjuvant S-1 chemotherapy (10–1). A relevant study reported no difference between serum levels of IGF1, IGF2, and IGFBP3 in stomach cancer cases and matched controls [16]. Another study in Korea examined the change in serum IGF1 and IGF2 levels in 20 stomach cancer cases after surgery using blood samples obtained within 10 days before and once after surgery [17]. The serum concentrations of IGF1 and IGF2 were significantly lower after surgery, but both pre- and postoperative serum concentrations were higher than those of age- and sex-matched controls. A Japanese study reported that two IGF1 SNPs (rs1520220 and rs2195239) were significantly associated with relapse-free survival in gastric cancer patients who had undergone curative gastrectomy [9]. In addition, an IGF1 gene polymorphism (rs5742612) was not associated with clinicopathological features in Iranian gastric cancer patients [18]. These data suggest that IGF- axis gene polymorphisms may be associated with gastric cancer progression, and that these associations may be modified by the cancer stage.

Hyperactivation of the IGF1R pathway by IGF1 has been associated with resistance to several chemotherapeutics, particularly cisplatin and etoposide, through continued activation of phosphoinositol-3-kinase signaling [19, 20]. In one study, genetic variants in IGFBP3 influenced the survival of patients with AGC treated with palliative chemotherapy [10]. The rs2854744 A allele and the rs2960436 A allele showed favorable associations with survival. In another study, chemorefractory wild-type KRAS metastatic colorectal cancer patients harboring the IGF1 rs2946834 variant A/A genotype had a significantly higher response rate to cetuximab (50%) compared to those with the A/G genotype (0%) [21].

The FOLFOX regimen is an effective palliative treatment for AGC [4, 5]. We reported on the effectiveness of oxaliplatin with biweekly low doses of leucovorin and bolus/continuous infusion of 5-FU (modified FOLFOX 4) as a first-line therapy in advanced gastric cancer patients and found a response rate of 50.0%, a median TTP of 7.7 months, and a median OS duration of 11.2 months [4]. Identification of patients with a potentially poor prognosis after FOLFOX chemotherapy would enable optimization of an alternative treatment protocol for patients with AGC. We previously evaluated the serum levels of IGF1 and their association with prognosis in patients with AGC who underwent FOLFOX chemotherapy. However, we did not demonstrate any statistically significant association between IGF1 and clinical outcomes [11]. In this study, we assessed 10 polymorphisms of the IGF-axis genes and their associations with response and survival in AGC patients treated with FOLFOX. To our knowledge, this is the first study to demonstrate a relationship between SNPs in the IGF-axis gene and response to FOLFOX chemotherapy in patients with AGC.

At least one SNP in IGF1R (rs12423791) and three SNPs in IGF1 (rs2162679, rs5742612, rs35767) were associated with tumor response to chemotherapy. Also, each SNP in IGFBP1 (rs4619) and IGF1R (rs17847203) were related to PFS, and five SNPs in IGF1R (rs7166558, rs2229765, rs12437963, rs2872060, and rs17847203) were significantly associated with OS, in univariate analyses. In a multivariate analysis, IGFB1 rs4619 (HR 0.575, 95% CI 0.385–0.858), and IGF1R rs17847203 (HR 2.530, 95%CI 1.289–4.966) were significantly correlated with PFS, and two IGF1R polymorphisms [rs2872060 and rs17847203; HR, 1.708 (95% CI, 1.024–2.850) and 2.087 (95% CI, 1.070–4.069), respectively] were related to OS in AGC patients treated with FOLFOX. However, none of the IGFBP3, IGFBP5, IRS1, IRS2 and IRS4 SNPs showed a statistically significant association with OS, which may be due to the limited number of patients analyzed.

Several previous studies assessed these SNPs in association with different types of cancer. Genetic variation in IGF2 and IFGBP3 may influence the risk of endometrial cancer in Caucasians, but IGFBP1 SNP rs4619 showed no such association [22]. No correlation between rs17847203 and its expression was found in adrenocortical tumors [23]. A study of genetic variations across IGF1R SNPs and the risk of breast cancer risk in Korean females showed that among 51 IGF1R SNPs, 5 intron-located SNPs (rs8032477, rs7175052, rs12439557, rs11635251, and rs12916884) were associated with a decreased risk of breast cancer [24]. However, we failed to find any significant associations between these SNPs and clinical outcomes in AGC treated with FOLFOX. The lack of such an association could be due to the diverse genetic background, different SNPs, the study population, and the chemotherapy regimen.

There were several limitations to this study. First, 9 of 53 SNPs were excluded from further analysis due to failure of genotyping, which may have resulted in loss of information. However, this was a technical limitation and may have led to selection bias. Second, a limited number of genes and SNPs were examined, creating a risk of potential false-positive findings related to multiple comparisons. Third, the retrospective design and relatively small numbers of patients involved in the present translational analysis indicate that the results should be considered hypothesis-generating and confirmed in prospective randomized controlled clinical trials.

Nevertheless, the study findings provide supporting evidence for the importance of genes of the IGF axis in AGC. From a clinical perspective, host genetic variants that are associated with IGF axis genotype do not simply indicate a new prognostic marker. Further, functional analysis of relationships between the significant SNPs and clinical features should be performed. Recently, a phase II/III trial of dalotuzumab and anti IGF1R monoclonal antibody, with standard treatment as a salvage therapy in metastatic colorectal cancer was reported [25]. That study suggested that the expression of IGF1 mRNA is a promising biomarker for anti-IGR1R therapies. Moreover, an SNP located in the 3′-untranslated region of the IGF1R gene may alter microRNA regulation of IGF1R expression [26]. The variant allele may reduce IGF1R expression and so be related to a poor response to anti-IGF1R treatment or chemotherapy. There remains a critical need to define predictive biomarkers to identify patients who may benefit from IGF1R-directed therapies or chemotherapy. We cannot conclude that our findings will facilitate selection of patients for IGF1-targeted therapy, which was recently evaluated in several types of cancer [27]. Therefore, it will be of interest to establish whether tumor IGF axis polymorphisms represent a favorable predictive profile for treatment with anti-IGF1R therapy in a larger patient population. AGC patients carrying the variant alleles associated with a poor clinical outcome might also show a poor response to targeted agents. Due to the small number of patients in this study, future independent validations in larger populations are necessary.

## MATERIALS AND METHODS

### Study population

All patients in this study had histologically confirmed adenocarcinoma of the stomach. These patients were treated by FOLFOX chemotherapy. All patients were ≥ 18 years of age, had an Eastern Cooperative Performance Status ≤ 2, and adequate organ function. Previous adjuvant chemotherapy was completed at least 6 months before inclusion. Exclusion criteria included the presence of central nervous system metastases, serious or uncontrolled concurrent medical illness, diabetes mellitus, and a history of other malignancies. Written informed consent was obtained from each patient before study entry. The Institutional Review Board of Dong-A University Hospital approved the use of all patient materials.

### Treatment protocols and assessment of response

On day 1, oxaliplatin (85 mg/m^2^) was administered by intravenous (i.v.) infusion in 500 ml of normal saline or dextrose over 2 h. On days 1 and 2, leucovorin (20 mg/m^2^) was administered as an i.v. bolus, immediately followed by 5-FU (400 mg/m^2^) given as a 10 min i.v. bolus, followed by 5-FU (600 mg/m^2^) as a continuous 22 h infusion with a light shield. Treatment was continued until there were signs of disease progression, unacceptable toxic effects developed, or the patient refused further treatment. The responses were evaluated using the RECIST criteria (version 1.1) [28].

### Measurements of serum levels of IGF1

A blood sample was drawn from each participant through venipuncture before chemotherapy. The blood samples were centrifuged for 10 min at 3,000 r/min at −4°C. The serum was subsequently removed and stored at −80°C until biochemical analysis. Serum IGF1 enzyme-linked immunosorbent assay (ELISA) was completed as per the manufacturer's protocol (R&D Systems, Minneapolis, MN, USA). Briefly, serum samples were thawed on wet ice 3 h prior to assay. IGF1 serum samples were pretreated with an acidic solution to promote dissociation of IGF1 from IGF1-binding proteins and stabilized in buffer containing preservatives. Samples were plated in duplicate in wells of a 96-well dish, after which a horseradish peroxidase-conjugated anti-IGF1 polyclonal secondary antibody was added. Substrate solution (tetramethylbenzidine in hydrogen peroxide) was then added and incubated for 30 min, following which the reaction was quenched with sulfuric acid. Plates were read at an absorbance of 450 nm on a Victor 3 plate reader (Perkin Elmer, Boston, MA, USA). Extrapolated absorbance was analyzed using the Masterplex Readerfit ELISA software (Hitachi, Waltham, MA, USA) and concentration was determined following a four-parameter logistic curve fit as per the manufacturer's recommendation. Measurements were performed by a single investigator blinded to the patients' clinicopathological data.

### DNA extraction and sample preparation

Blood collected from each enrolled patient before chemotherapy onset was used for genotyping. DNA was automatically extracted from the 75 μL of the buffy coat layer using the MagAttract DNA Blood Midi M48 Kit (Qiagen, Inc., Valencia, CA), and a Qiagen BioRobot M48 workstation, according to the manufacturer's protocols. The purity and concentration of isolated DNA were determined using a ND-1000 spectrophotometer (Nanodrop Technologies, Wilmington, DE, USA). Since accurate information regarding the quantity of each sample was necessary for genotyping, the quantity of DNA was measured using a Quant-iT^™^ PicoGreen^®^ dsDNA Assay Kit (Molecular Probes, Inc., Eugene, OR, USA) using dry plates for the genotyping reaction and 10 ng DNA in each well of 384-well plates.

### Genotyping

The SNPs have been described previously [9, 14]. The multiplexed assay group was designed to test 53 SNPs using a MassARRAY Assay Designer v3.0 (Sequenom, San Diego, CA, USA) and genotyped (detailed information for selected SNPs and assay design in Supplementary Tables 1 and 2). Genotyping was carried out using the iPLEX Gold^™^ assay on the MassARRAY^®^ Platform (Sequenom). PCR reactions were performed in a total volume of 5 μL with 10 ng of genomic DNA, 1.625 mM MgCl2, 0.1 unit of HotStarTaq polymerase (Qiagen), 0.5 mM dNTP (Invitrogen, Inc., Carlsbad, CA, USA), and 100 nM primers. PCR commenced at 94°C for 15 min, followed by 45 cycles at 94°C for 20 s, 50°C for 30 s, and 72°C for 1 min, with the final extension at 72°C for 3 min. Amplified PCR products were treated by a mixture shrimp alkaline phosphatase (SAP) in 7 μL buffer. The SAP reaction proceeded at 37°C for 40 min and then 85°C for 5 min. The regions containing target SNPs were amplified by PCR and treated with SAP followed by a single-base extension reaction, resulting in an allele-specific difference in mass between extension products. The extension reactions were performed in a total volume of 9 μL with 50 μM dNTP/dideoxynucleotide phosphate (ddNTP) each, 0.063 units/μL Thermo Sequenase (both from Sequenom), and 625 nM to 1.25 μM extension primers. Under the cycling conditions, two cycling loops, one of five cycles inside a loop of 40 cycles, were used. The sample was denatured at 94°C. Strands were annealed at 52°C for 5 s and extended at 80°C for 5 s. The annealing and extension cycle was repeated a further four times for a total of five cycles and then looped back to the 94°C denaturing step for 5 s, after which the five-cycle annealing and extension loop was conducted again. The five annealing and extension steps and the single denaturing step were repeated a further 39 times for a total of 40 cycles. The 40 cycles of the five-cycle annealing and extension steps equated to a total of 200 cycles (5 × 40). A final extension was performed at 72°C for 3 min and then the sample was cooled to 4°C. After cleaning up the extension reaction products with SpectroCLEAN, the products were transferred to SpectroCHIP using SpectroPOINT and then processed using a SpectroREADER matrix-assisted laser desorption/ionizationtime of flight (MALDI-TOF) spectrometer. Resulting genotype data were collected by Typer v4.0 (Sequenom) and genotype clusters were examined manually for their fitness.

### Statistical analyses

Serum levels of IGF1 are expressed as means ± standard deviation. Associations between IGF axis SNPs and levels of serum IGF1 were assessed by Kruskal-Wallis test. The association between IGF-axis SNPs and response to chemotherapy was assessed by chi-squared tests. The genetic model of inheritance for IGF1 and IGF1R polymorphisms was unknown, so we considered the dominant, recessive, co-dominant, or additive model, as appropriate. All SNPs were examined for deviation from Hardy–Weinberg equilibrium (HWE) by comparing actual allelic distributions with those expected from HWE using a chi-squared test. Linkage disequilibrium among polymorphisms in the IGF axis was assessed using D′ and r2 values, and the haplotype frequencies of the two genes were inferred using the Haploview version 4.2 software (Broad Institute, Cambridge, MA, USA) [29]. The primary end point of the study was the associations between genotypes and overall survival (OS). Progression-free survival (PFS) and OS were calculated from the date of initiation of therapy to the date of disease progression or death. Patients who were alive at the last follow-up were censored at that time. Patients who were excluded from this study or who died before disease progression were screened at the time of exclusion from this study. The association of each SNP with survival was analyzed using Kaplan–Meier plots and the log-rank test, and the associated 95% confidence intervals (CIs) were calculated. Hazard ratios (HRs) for PFS or OS, together with their 95% CIs, were calculated using multivariate Cox proportional hazards regression. All tests were two-sided, and *P* < 0.05 was considered to indicate statistical significance. Statistical analyses were performed using IBM SPSS Statistics version 20.0.

## CONCLUSIONS

These data provide the first evidence that genetic polymorphisms within the IGF axis are significantly associated with chemotherapy response, PFS, and OS in AGC patients treated with FOLFOX. This information is not simply related to a novel prognostic marker, but rather, it is strictly related to the possible development and optimization of target therapies that exploit the IGF pathway. Further validation in larger cohort or independent population and functional characterizations are needed.

## SUPPLEMENTARY MATERIAL TABLES






