# Supplementary material for: Relationship between insulin-like growth factor axis gene polymorphisms and clinical outcome in advanced gastric cancer patients treated with FOLFOX
Source: Oncotarget. 2016 Apr 29;7(21):31204–14. doi: 10.18632/oncotarget.9100 (PMC5058750; doi:10.18632/oncotarget.9100)
Supplement: Supplementary file 1 [file oncotarget-07-31204-s001.pdf]

Relationship between insulin-like growth factor axis gene polymorphisms and clinical outcome in advanced gastric cancer patients treated with FOLFOX

Supplementary Materials

Supplementary Table S1: Analyzed SNPs of IGF axis genes. See Supplementary_Table_S1

Supplementary Table S2: Primer sequences for SNPs of IGF axis genes. See Supplementary_Table_S2

Supplementary Table S3: Combination of risk alleles

No of risk allele*	No. of patients	HR for PFS (95 CI)	*P* value
1	24	1.0	
2	19	0.64 (0.09–4.17)	0.643
3	128	0.65 (0.18–2.41)	0.529
4	10	1.21 (0.12–11.81)	0.865

HR, hazard ratio; PFS, progression free survival; CI, confidence interval;
*Risk alleles: A for rs7166558, G for rs2229765, G for rs12437963, T/T for rs2872060 and T for rs17847203.
