# Supplementary material for: Relationship between insulin-like growth factor axis gene polymorphisms and clinical outcome in advanced gastric cancer patients treated with FOLFOX
Source: Oncotarget. 2016 Apr 29;7(21):31204–14. doi: 10.18632/oncotarget.9100 (PMC5058750; doi:10.18632/oncotarget.9100)
Supplement: Supplementary file 2 [file oncotarget-07-31204-s002.doc]

Supplementary Table S1: Analyzed SNPs of IGF axis genes
										
rs11042751	chr11:2149864	11p15.5		A/G	IGF2	Downstream	-	-	fail	
rs734351	chr11:2156213	11p15.5	6349	C/T	IGF2	Intron	-	-		
rs10860862	chr12:102786072	12q23.2	3285467	G/T	IGF1	Downstream	-	-		
rs2946834	chr12:102787814	12q23.2	1742	C/T	IGF1	Downstream	-	-		
rs5742714	chr12:102789852	12q23.2	2038	C/G	IGF1	3′ UTR	-	-	design fail	
rs6219	chr12:102790192	12q23.2	340	A/G	IGF1	3′ UTR	-	-		
rs1063599	chr12:102792569	12q23.2	2377	A/G	IGF1	3′ UTR	-	-	fail	
rs6214	chr12:102793569	12q23.2	1000	A/G	IGF1	3′ UTR	-	-		
rs1520220	chr12:102796522	12q23.2	2953	C/G	IGF1	Intron	-	-		
rs6539035	chr12:102800935	12q23.2	4413	C/T	IGF1	Intron	-	-		
rs4764887	chr12:102823900	12q23.2	22965	A/G	IGF1	Intron	-	-		
rs2288378	chr12:102830008	12q23.2	6108	A/G	IGF1	Intron	-	-		
rs10735380	chr12:102844236	12q23.2	14228	A/G	IGF1	Intron	-	-		
rs2195239	chr12:102856702	12q23.2	12466	C/G	IGF1	Intron	-	-		
rs7956547	chr12:102858816	12q23.2	2114	C/T	IGF1	Intron	-	-		
rs12423791	chr12:102858828	12q23.2	12	C/G	IGF1	Intron	-	-		
rs2162679	chr12:102871259	12q23.2	12431	A/G	IGF1	Intron	-	-		
rs5742612	chr12:102874864	12q23.2	3605	C/T	IGF1	Promoter	-	-		
rs35767	chr12:102875569	12q23.2	705	C/T	IGF1	Promoter	-	-		
rs2289046	chr13:110407906	13q34	2428391	A/G	IRS2	3′ UTR	-	-		
rs1974134	chr13:110428702	13q34	20796	C/T	IRS2	Intron	-	-	fail	
rs7981705	chr13:110431891	13q34	3189	C/T	IRS2	Intron	-	-		
rs9521511	chr13:110434223	13q34	2332	A/G	IRS2	Intron	-	-		
rs1805097	chr13:110435231	13q34	1008	A/G	IRS2	Coding exon	G/D	1057	fail	
rs12853546	chr13:110435914	13q34	683	A/G	IRS2	Coding exon	P/P	829	fail	
rs8041224	chr15:99297665	15q26.3	53336590	C/T	IGF1R	Intron	-	-		
rs2684761	chr15:99364370	15q26.3	66705	A/G	IGF1R	Intron	-	-		
rs1879612	chr15:99398708	15q26.3	34338	C/T	IGF1R	Intron	-	-		
rs3743262	chr15:99465473	15q26.3	66765	C/T	IGF1R	Coding exon	T/T	766		
rs4966044	chr15:99466198	15q26.3	725	A/G	IGF1R	Intron	-	-		
rs7166558	chr15:99475484	15q26.3	9286	A/G	IGF1R	Intron	-	-		
rs2229765	chr15:99478225	15q26.3	2741	A/G	IGF1R	Coding exon	E/E	1043		
rs2684799	chr15:99481832	15q26.3	3607	A/G	IGF1R	Intron	-	-		
rs12437963	chr15:99496859	15q26.3	15027	A/G	IGF1R	Intron	-	-		
rs2872060	chr15:99499493	15q26.3	2634	G/T	IGF1R	Intron	-	-		
rs17847203	chr15:99500605	15q26.3	1112	C/T	IGF1R	Coding exon	Y/Y	1346		
rs11575194	chr2:217543728	2q35	57026247	C/T	IGFBP5	Coding exon	R/W	138	monomorphic	
rs7420849	chr2:217550529	2q35	6801	G/T	IGFBP5	Intron	-	-		
rs35802832	chr2:227611915	2q36.3	10061386	C/T	IRS1	Intron	-	-		
rs1025333	chr2:227645525	2q36.3	33610	A/T	IRS1	Intron	-	-	monomorphic	
rs2288586	chr2:227655390	2q36.3	9865	C/G	IRS1	Intron	-	-		
rs1801278	chr2:227660544	2q36.3	5154	A/G	IRS1	Coding exon	G/R	971	fail	
rs1801276	chr2:227661921	2q36.3	1377	C/G	IRS1	Coding exon	A/P	512	monomorphic	
rs8191754	chr6:160448324	6q25.3	50012410	C/G	IGF2R	Coding exon	L/V	252		
rs1570070	chr6:160453978	6q25.3	5654	A/G	IGF2R	Coding exon	S/S	350		
rs998075	chr6:160468278	6q25.3	14300	C/T	IGF2R	Coding exon	T/T	713		
rs1803989	chr6:160517481	6q25.3	49203	C/T	IGF2R		-	-		
rs4619	chr7:45932669	7p12.3	43776456	A/G	IGFBP1	Coding exon	I/M	253		
rs6670	chr7:45952254	7p12.3	19585	A/T	IGFBP3	3′ UTR	-	-		
rs9282734	chr7:45956969	7p12.3	4715	A/C	IGFBP3	Coding exon	H/P	164		
rs2854746	chr7:45960645	7p12.3	3676	C/G	IGFBP3	Coding exon	A/G	32	fail	
rs2854744	chr7:45961075	7p12.3	430	A/C	IGFBP3	Promoter	-	-		
rs2073115	chrX:107979515	Xq22.3	5103946	C/T	IRS4	Coding exon	A/A	20	design fail	
SNP, single nucleotide polymorphism; RS#, reference SNP identification number; UTR, untranslated region.	
