# Supplementary material for: Relationship between insulin-like growth factor axis gene polymorphisms and clinical outcome in advanced gastric cancer patients treated with FOLFOX
Source: Oncotarget. 2016 Apr 29;7(21):31204–14. doi: 10.18632/oncotarget.9100 (PMC5058750; doi:10.18632/oncotarget.9100)
Supplement: Supplementary file 3 [file oncotarget-07-31204-s003.doc]

Supplementary Table S2: Primer sequences for SNPs of IGF axis genes
SNP rs#	Forward primer (5′ → 3′)	Reverse primer (5′ → 3′)	Extension primer (5′ → 3′)	
rs11042751	ACGTTGGATGCCTTCCAAGG
TTGAGGCTG	ACGTTGGATGTTGGGACAGGTG
GGTACAG	GGAGGCAGTGGTACTAA	
rs734351	ACGTTGGATGGAAGAGCCT
GCCTTTCTTCC	ACGTTGGATGTCTGATCATTCA
CCGAACGC	gTTCCTGCACCTTTTGGA
CATT	
rs10860862	ACGTTGGATGGATGGAGAA
GCACAATCCTG	ACGTTGGATGAGGTGATTGTTC
CTGGAGAG	GCACAATCCTGGGTCGT	
rs2946834	ACGTTGGATGCATGCACAT
GTGGAAGAACG	ACGTTGGATGGATAAAGAAGCA
TTTCTGCAC	tTGGGGAATGACACATTATT	
rs6219	ACGTTGGATGTGCTAAACA
CACTGCAGGAG	ACGTTGGATGTTCATGATACAC
AGACACAG	GAGGGACTCTGAAACCTC
AA	
rs1063599	ACGTTGGATGAAAAATATG
TCTATGCTTTC	ACGTTGGATGCCAGCTATGCCA
ATGTGGTG	cccacATGTCTATGCTTTCTTT
TTTTT	
rs6214	ACGTTGGATGACTTTATAG
GCAGTCTTCTG	ACGTTGGATGGACACTCTTAG
AATTATCAC	cctgaAGTCTTCTGCAGACT
TAAC	
rs1520220	ACGTTGGATGGGGTACAT
AGAAGATGCCTG	ACGTTGGATGCCTGAGAAGG
GCATGTATAG	AGAAGATGCCTGAGTGAG
GTTTGG	
rs6539035	ACGTTGGATGAAAGATC
ACTGAGGACTAA	ACGTTGGATGGTAAACTGTA
AAGCCTTTGC	aaggTCACTGAGGACTAATA
ACAA	
rs4764887	ACGTTGGATGCTCTTTG
TCTCACATCTGGG	ACGTTGGATGAGAGCAGAGCT
ACTTATGCC	GGTTTGAGAAAGCTGTGT	
rs2288378	ACGTTGGATGACCTTTT
TCCCTTCCCTGAG	ACGTTGGATGTGGCTTGTTGT
CTTCAGGTG	TTTGCACAGTCTGTGTCCTT	
rs10735380	ACGTTGGATGCACGAAT
GGAAACAGCCATC	ACGTTGGATGGTCGAATTTAG
TCCCAGAGC	AACAGCCATCGTACACT	
rs2195239	ACGTTGGATGACTCACA
GTGAAATGGTTGG	ACGTTGGATGGCTATTGAGTGA
AAAAACAGG	GGTTGGATCCATAGGAAG	
rs7956547	ACGTTGGATGTCTTCAG
AATGCTCACTCAG	ACGTTGGATGGTGGTAAAATG
ATAGGCCCC	ttGAATGCTCACTCAGTAT
CACT	
rs12423791	ACGTTGGATGCTGCTTC
TTCCAATGAGAGG	ACGTTGGATGACATTGAGACC
ACCCAAGAG	AGAGGAAATAGTGATACTGA	
rs2162679	ACGTTGGATGTTTCTCA
ACAAGATCTCCGC	ACGTTGGATGAACATGCTTC
TTCCAGTCCC	ATGGAAATCTTCCACCC	
rs5742612	ACGTTGGATGCCTCCAT
AGGTTCTAGGAAA	ACGTTGGATGAGATTGGAAGA
CAGCACTCG	gcgAGGAAATGAGATCACACC	
rs35767	ACGTTGGATGTCAGTTG
ATGTGTCAGTCCC	ACGTTGGATGGTGGTGGAAA
TAACCTGGAC	AGTCCCCTGAGAGTCATG	
rs2289046	ACGTTGGATGTCCCACC
TCCCACTACCCA	ACGTTGGATGGGATTGGACTT
TGAAGACGG	CCTCCCACTACCCAATACAG	
rs1974134	ACGTTGGATGTTATGTG
TCCAAAGACACTG	ACGTTGGATGCATAAGAAAAC
TGGACAGAG	cTAAAACTTGAATGGGTA
AAGG	
rs7981705	ACGTTGGATGGGGTATT
TATCCCAAGTTCC	ACGTTGGATGTGTCACCTTAG
TCCACGTAG	CCTAAATGCTTCCTTTCTG
TCTCAA	
rs9521511	ACGTTGGATGCTCCAAG
ACGAATGTTCCTG	ACGTTGGATGGGCACTGGCTC
CTTGTTTTG	ACGAATGTTCCTGGCAAGTT
CTCTGA	
rs1805097	ACGTTGGATGTGTCCGA
GGACAACGATGAG	ACGTTGGATGGGAGCTGTACC
GCCTGCCC	agagtACAACGATGAGGC
GGCG	
rs12853546	ACGTTGGATGAGACGCT
CCTCCTCCAGGAT	ACGTTGGATGTACAAGGCCCC
CTACACCTG	CGTGCTCATGAGCTCCCC	
rs8041224	ACGTTGGATGTCACAAC
CACAACACCAACC	ACGTTGGATGTCTGCCTGTGA
GTATTTGAG	CAGCTTCTCAACTGGTA
CTTAAA	
rs2684761	ACGTTGGATGGTTTACACA
CTCTTGGACGC	ACGTTGGATGGTAGATGAACTA
GAAGATGG	CGCCTGAGTTCTCCTTGA	
rs1879612	ACGTTGGATGCTGTAATTC
TTTCTGCAGTCC	ACGTTGGATGGCAGGTACTTAGG
AAGTGAG	agggaTTTCTGCAGTCCTATA
GC	
rs3743262	ACGTTGGATGTCATGCAA
GTGGCCAACACC	ACGTTGGATGTACTCTGTCTCC
AGCTCTTC	CGCAGACACCTACAACAT
CAC	
rs4966044	ACGTTGGATGTCCATCTTT
GAAGGTGGGTG	ACGTTGGATGTGCTCTTTCTA
CCACCTGAG	ggCTTCAAAGACAGAACAA
GA	
rs7166558	ACGTTGGATGTAAGTGCT
TCTCAAAGGCCG	ACGTTGGATGAACTTATTGCCA
CCCAGGTC	tgtgaTTCTCAAAGGCCGAATA
AAT	
rs2229765	ACGTTGGATGCAGAAGCT
TCGTTGAGAAAC	ACGTTGGATGTAAAACAGTGA
ACGAGGCCG	CTTCGTTGAGAAACTCAAT
CCT	
rs2684799	ACGTTGGATGTTCAGCTGG
ACTTCAGTTGC	ACGTTGGATGTTTCACCTTAC
AATGTGGC	tcttaAGTTGCCAAGGACTTGT
CTTC	
rs12437963	ACGTTGGATGTGTGGAAG
TGTTAGCAGCAG	ACGTTGGATGTGAACGCAGCC
GTGGAAGAC	AAGTGTTAGCAGCAGAACA
AATGGCT	
rs2872060	ACGTTGGATGAGAACAGG
ATGACGCTTGTG	ACGTTGGATGCTCACAGGTAT
TTGCAGCCC	ccGACGCTTGTGCTGTGTG
TCGGT	
rs17847203	ACGTTGGATGTTCTTGCGG
CCCCCGTTCAT	ACGTTGGATGGGGGTGCTGG
TCCTCCGC	ttttCCCCCGTTCATGTGGGC	
rs11575194	ACGTTGGATGAGATGGCC
GAGGAGACCTAC	ACGTTGGATGTCTTCACTGC
TTCAGCCTTC	ACTCCCCCAAGATCTTC	
rs7420849	ACGTTGGATGTGCTGGTC
AGAGATGGGATG	ACGTTGGATGAAGGTGCTG
TGGACACTTC	cCAGAGATGGGATGGGTAG
GAAAAG	
rs35802832	ACGTTGGATGGGCGGTTC
CTTAGCTAAAAG	ACGTTGGATGTCTGGCTGAG
CTAAAAGCAC	aTTAGCTAAAAGTTGTCA
CTACCT	
rs1025333	ACGTTGGATGCTCAAACC
ATTTCATTTGTCC	ACGTTGGATGCATACCAGATTA
CAGTCTTG	cccCCATTTCATTTGTCCA
ATATTT	
rs2288586	ACGTTGGATGAGCATTT
CTCACCAGCCAAC	ACGTTGGATGACTTGGCCCCTC
AGAAAAAG	gCCAGCCAACATCCCTCTTG	
rs1801278	ACGTTGGATGATGGTCA
TGTAGTCACCCCG	ACGTTGGATGTCGAGATGGG
CAGACTGGG	GTAGGCCTGCAAATGCTAG
CAGCCC	
rs1801276	ACGTTGGATGGTGAGTT
CTCTTTCGGAACC	ACGTTGGATGAACAGGCTTGG
GCACGAGTC	ccccaCAGATCTGCAGCAC
TGG	
rs8191754	ACGTTGGATGAAAAACG
CCAACAGCATCGG	ACGTTGGATGACACCAGGCGT
TTGATGTTG	CTTGCGCACCAGCTTCA	
rs1570070	ACGTTGGATGCAGTTTCT
CCACAGACATTC	ACGTTGGATGGCCTCTTCTT
GTTAATTTCCC	gCTTTTCCATCTGAAATA
TAGGA	
rs998075	ACGTTGGATGATCCAACT
GAACTACAGAGG	ACGTTGGATGACAGAGAAA
GGTGATGAGCG	CAACTGAACTACAGAGGC
GGCAC	
rs1803989	ACGTTGGATGTTCCGCAC
TTAGAGGAAGAG	ACGTTGGATGATGGTGACA
TGCCATGTGTG	ggagGAGGCAAACACGA
CATC	
rs4619	ACGTTGGATGTACCCTTG
GAATGGGAAGAG	ACGTTGGATGTCATCTGGT
TTCAGTTTTG	aggGACCCCAACTGCCA
GAT	
rs6670	ACGTTGGATGTTCCTCAG
TCATGGCCACAG	ACGTTGGATGCAACATAGC
CCCAAATATAG	CATGGCCACAGTTGTATCA	
rs9282734	ACGTTGGATGAAATGCTA
GTGAGTCGGAGG	ACGTTGGATGGGTGGAAC
TTGGGATCAGAC	GCCCGTCCGTCTCCAGC
ACGC	
rs2854746	ACGTTGGATGTGACTCT
GCTGGTGCTGCTC	ACGTTGGATGTCGCAGCG
CACCACGGGAC	tGGCTGGCGCGAGCTCGG	
rs2854744	ACGTTGGATGGGTTCTT
GTAGACGACAAGG	ACGTTGGATGGTGCAGCT
CGAGACTCGCC	cCCCGGGCTCCGGGCGTG	
SNP, single nucleotide polymorphism; RS#, reference SNP identification number.	
